# Modeling and Simulation for Predicting Thermo-Mechanical Behavior of Wafer-Level Cu-PI RDLs During Manufacturing

**DOI:** 10.3390/mi16050582

**Published:** 2025-05-15

**Authors:** Xianglong Chu, Shitao Wang, Chunlei Li, Zhizhen Wang, Shenglin Ma, Daowei Wu, Hai Yuan, Bin You

**Affiliations:** 1 Pen-Tung Sah Institute of Micro-Nano Science and Technology, Xiamen University, Xiamen 361005, China; 2 31002 PLA Troops, Beijing 100161, China; 3 Xi’an Microelectronic Technology, Xi’an 710071, China; 4 92858 PLA Troops, Ningbo 315800, China

**Keywords:** equivalent model, redistribution layer, wafer-level model, thermo-mechanical, polyimide, layout effect

## Abstract

The development of chip manufacturing and advanced packaging technologies has significantly changed redistribution layers (RDLs), leading to shrinking line width/spacing, increasing the number of build-up layers and package size, and introducing organic materials such as polyimide (PI) for dielectrics. The fineness and complexity of structures, combined with the temperature-dependent and viscoelastic properties of organic materials, make it increasingly difficult to predict the thermo-mechanical behavior of wafer-level Cu-PI RDL structures, posing a severe challenge in warpage prediction. This study models and simulates the thermo-mechanical response during the manufacturing process of Cu-PI RDL at the wafer level. A cross-scale wafer-level equivalent model was constructed using a two-level partitioning method, while the PI material properties were extracted via inverse fitting based on thermal warpage measurements. The warpage prediction results were compared against experimental data using the maximum warpage as the indicator to validate the extracted PI properties, yielding errors under less than 10% at typical process temperatures. The contribution of RDL build-up, wafer backgrinding, chemical mechanical polishing (CMP), and through-silicon via (TSV)/through-glass via (TGV) interposers to the warpage was also analyzed through simulation, providing insight for process risk evaluation. Finally, an artificial neural network was developed to correlate the copper ratios of four RDLs with the wafer warpages for a specific process scenario, demonstrating the potential for wafer-level warpage control through copper ratio regulation in RDLs.

## 1. Introduction

As the density and performance of interconnects continue to increase with the development of integrated circuits, advanced packaging technologies—such as CoWoS [1] and Foveros [2]—have become key enablers in extending beyond the traditional limits of Moore’s Law. The RDL, the core structure for package-level interconnection, provides electrical routing between chips and substrates through its multi-layer metal wiring, thereby enhancing the interconnect density and signal transmission efficiency. Due to their excellent electrical conductivity and compatibility with fine-pitch interconnects, thick copper damascene structures were first adopted in back-end packaging RDLs such as TSMC’s CoWoS and Amkor’s SWIFT [3]. However, the high stiffness and residual stress present significant warpage control and reliability challenges for large-sized wafer-level or panel-level packaging with thick copper damascene structures. Therefore, the industry has shifted toward organic dielectric materials with a higher patterning resolution, such as polyimide (PI), and adopted damascene or SAP (semi-additive process) techniques to build organic-based RDLs as alternative solutions [4,5,6,7], replacing conventional inorganic dielectrics such as silicon dioxide (SiO_2_) and silicon nitride (Si_3_N_4_). PI is particularly suitable for multi-die or high-aspect-ratio packaging applications with its relatively low elastic modulus and superior stress relief capability.

The manufacture of RDLs usually involves high-temperature processes (typically ranging from 200 °C to 250 °C) for RDL build-up, coupled with factors such as coefficient of thermal expansion (CTE) mismatch between different materials and residual stress, which will inevitably lead to warpage and increase risks of damage. This problem is significant in wafer-level and panel-level manufacturing processes. Warpage may result in process problems such as alignment deviation [8,9,10], reduced bonding quality [11,12,13,14], and vacuum adsorption failure [15,16]. These affect the yield of downstream processes and reduce the product’s reliability, making warpage one of the core challenges restricting the improvement of yield and cost reduction in advanced packaging.

Therefore, in recent years, how to effectively predict wafer-level and panel-level warpages to reduce process risks and cost consumption has become a research hotspot. Among them, the multi-physics field equivalent modeling and simulation method based on Finite Element Analysis (FEA) has attracted considerable attention in advanced packaging, since it significantly simplifies calculations while retaining key physical mechanisms [17,18,19]. In 2022, Lee et al. adopted a method that considers both CTE mismatch and interfacial mechanical responses to perform equivalent treatment on an RDL composed of Cu and a photo-imageable dielectric (PID) inside a fan-out panel-level package (FO-PLP). The panel-level warpage after the entire RDL process was predicted and validated [20]. In the same year, Unimicron directly converted a fine metal L/S/H RDL-first substrate into an equivalent homogeneous block based on the thickness of the metal layer and the dielectric layer. The warpage of the hybrid substrate with Ajinomoto Build-Up Film (ABF) was predicted [21]. In 2023, Wu et al. proposed a machine learning-based modeling and simulation method for the thermo-mechanical behavior prediction of TSV interposers and RDLs. A thermo-mechanical model preserving layout information for a 2.5D integrated CPU chip was developed by integrating artificial neural networks (ANNs) [22]. In 2025, Wu et al. used the representative volume element (RVE) equivalent method to homogenize each RDL inside the fan-out package-on-package (FOPoP) according to its copper proportion. They combined the “element birth and death” method to predict the wafer warpage in the FOPoP RDL manufacturing process [23]. As reviewed, most existing equivalent modeling methods either simplify the entire RDL as a uniform material in large-sized wafer-level and panel-level packages, or are only applicable to chip-level packages. However, as the RDL line width and spacing continue to shrink and the number of build-up layers and the overall package size increase, the impact induced by the layout of lines becomes non-negligible. Furthermore, organic dielectric materials like PI exhibit complex viscoelastic and temperature-dependent properties. These issues may lead to the failure of existing equivalent modeling methods and increase the difficulty in predicting the thermo-mechanical behavior of wafer-level or panel-level Cu-PI RDL structures under thermal stress. Therefore, when applying equivalent modeling approaches to wafer-level or panel-level Cu-PI RDLs with fine-pitch features, it is necessary to consider the complex material characteristics of Cu-PI and cross-scale modeling.

To address the above challenges, this study proposed a novel cross-scale modeling and simulation method that focused on the wafer-level thermo-mechanical behavior of Cu-PI RDLs during manufacturing. Unlike most existing works that have relied on small-scale or simplified structures, we developed a wafer-level equivalent modeling approach that explicitly preserved the real RDL layout features. This layout-aware modeling enabled both high prediction accuracy and computational efficiency for full-wafer warpage analysis. Specifically, a two-level partition-based homogenization strategy was proposed to construct the cross-scale equivalent model. Based on the thermal warpage measurements of a PI-layered wafer, the temperature-dependent material properties of PI were calibrated by inverse fitting. The fitting properties of PI were then validated using Cu-PI RDL wafer warpage simulations at various process temperatures. This layout-sensitive equivalent model was further used to investigate the warpage impact of RDL build-up, wafer backgrinding, CMP, and TSV/TGV interposers. Finally, taking the copper ratio (the copper fraction in a unit cell) distributions across four RDLs under the RDL build-up condition as an example, an artificial neural network (ANN) was trained to correlate with the wafer warpage, demonstrating the potential for wafer-level warpage control through copper ratio regulation in RDLs. This study provided a layout-aware, high-accuracy, and efficient wafer-level warpage prediction framework for Cu-PI RDL structures, supporting design and process co-optimization in advanced packaging.

## 2. Materials and Methods

### 2.1. Structure

**Figure 1:**
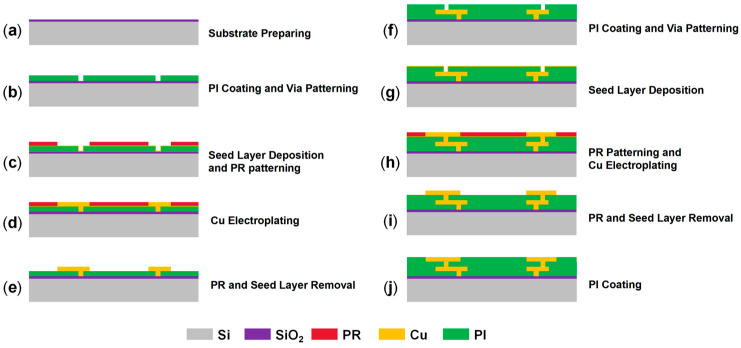
Fabrication process of RDL wafer with two metal layers and two via layers through SAP: (**a**) Substrate preparing. (**b**) PI coating and via etching. (**c**) Seed layer deposition and PR patterning. (**d**) Cu electroplating. (**e**) PR and seed layer removal. (**f**) PI coating and via etching again. (**g**) Seed layer deposition. (**h**) PR patterning and Cu electroplating. (**i**) PR and seed layer removal. (**j**) PI coating.

To validate the equivalent modeling and simulation method proposed in this work, an 8-inch RDL wafer (minimum line/spacing: 4 μm/4 μm) with two metal layers and two via layers was fabricated using the SAP process (Figure 1). The fabrication process began with substrate preparation, followed by PI (JSR ELPAC WPR-5100) spin-coating and via opening (Figure 1a,b). A Cu seed layer was then deposited by sputtering, and the photoresist (PR) was spin-coated and patterned (Figure 1c). Cu electroplating was subsequently performed to form one via layer and one metal layer (Figure 1d). After stripping the PR layer and removing the seed layer, another PI layer was spin-coated, and vias were opened (Figure 1e,f). The sequence—Cu seed layer deposition, PR layer coating and patterning, and Cu electroplating—was repeated to form the second via layer and the second metal layer (Figure 1g,h). Finally, the PR and seed layers were removed, and another PI layer was spin-coated and cured, resulting in an RDL wafer with two metal layers and two via layers (Figure 1i,j).

**Figure 2:**
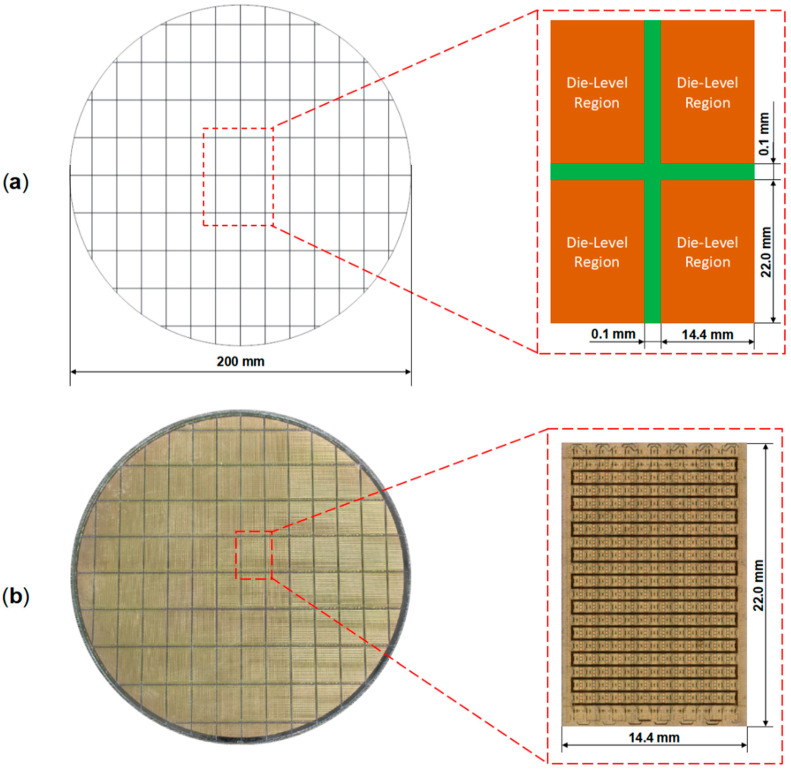
Top view of the 200 mm RDL wafer sample: (**a**) Die arrangement on the wafer with dummy structures at the edge. (**b**) Top view of single die-level region with layout structure.

Figure 2 shows the top view of the 8-inch RDL wafer sample. The die-level region has a planar dimension of 14.4 mm × 22.0 mm, with a scribe line width of 0.1 mm between adjacent die-level regions (Figure 2a). Each die-level region on the wafer has an identical structure, and the layout design of the die-level region can be seen in Figure 2b.

### 2.2. Equivalent Finite Element Model

When evaluating the impact of the RDL layout, directly modeling each RDL using the conformal method will face challenges such as high complexity, excessive mesh and node count, and low solving efficiency. A partitioned equivalent method, consisting of three levels—the block level, die level, and wafer level—was adopted to improve simulation efficiency while considering the RDL layout.

#### 2.2.1. Block-Level Model

**Figure 3:**
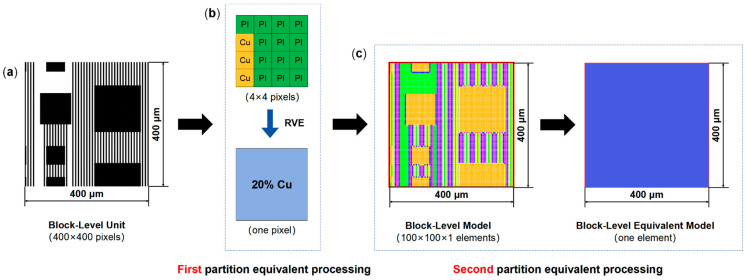
Two-level partition equivalent processing method for block-level model. (**a**) Block-level unit with 400 × 400 pixels. (**b**) First partition equivalent processing method. (**c**) Second partition equivalent processing method.

The modeling process commenced with developing a block-level equivalent model to simplify the final wafer-level equivalent model while considering RDL layout effects. Taking the block-level model of the bottom-most metal layer (M1 layer, i.e., the first metal layer from bottom to top) as an example, Figure 3 illustrates the two-level partition equivalent process method. Initially, the die-level layout CAD file of the M1 layer was converted into a PNG image (28,800 × 44,000 pixels) and subsequently segmented into block-level units with a planar size of 400 μm × 400 μm (400 × 400 pixels), as shown in Figure 3a. Metal coverage of each 4 × 4 pixel region inside the unit is supposed to be evaluated. For example, a 4 × 4 pixel region containing three metal pixels corresponds to an approximate metal coverage of 20%. This 4 × 4 pixel region was then simplified into a single pixel through the RVE (representative volume element) method, as shown in Figure 3b. By such simplification, each block-level unit was reduced to 100 × 100 pixels, which was then transformed into a metal coverage distribution matrix. A corresponding block-level finite element model (100 × 100 × 1 elements) was established in Ansys APDL 2024 R1. Subsequently, virtual finite element simulations were conducted to extract the equivalent thermo-mechanical material properties of the block-level model. After that, a block-level model of 10,000 elements was simplified to a block-level equivalent model of a single element (Figure 3c).

**Figure 4:**
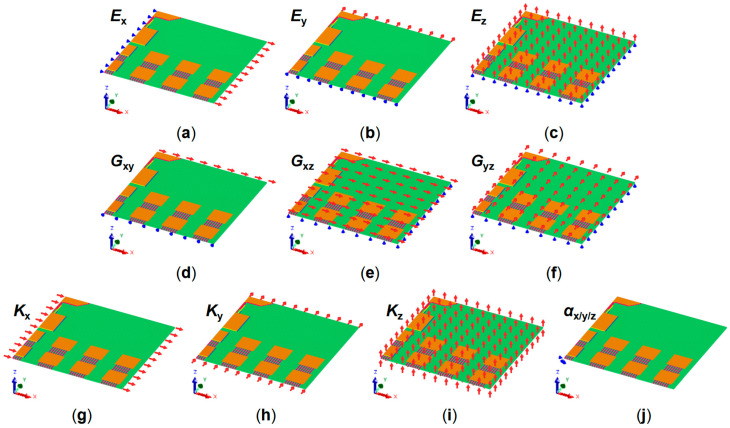
RDL block equivalent material properties calculation method. With different boundary conditions, the orthotropic material property of RDL block calculated in ANSYS APDL includes (**a**) Young’s modulus in X direction; (**b**) Young’s modulus in Y direction; (**c**) Young’s modulus in Z direction; (**d**) shear modulus in X-Y plane; (**e**) shear modulus in X-Z plane; (**f**) shear modulus in Y-Z plane; (**g**) thermal conductivity in X direction; (**h**) thermal conductivity in Y direction; (**i**) thermal conductivity in Z direction; (**j**) coefficient of thermal expansion in X/Y/Z directions.

The virtual finite element test boundary conditions used to calculate the equivalent thermo-mechanical material properties of the block-level model are shown in Figure 4. The block-level model was constructed using Ansys APDL 2024 R1 and meshed using hexahedral 8-node elements. The orthotropic material properties to be calculated include Young’s modulus $E_{x}$/$E_{y}$/$E_{z}$, Poisson’s ratio $\nu_{\mathrm{xy}}$/$\nu_{\mathrm{xz}}/\nu_{\mathrm{yz}}$, shear modulus $G_{\mathrm{xy}}$/$G_{\mathrm{xz}}/G_{\mathrm{yz}}$, thermal conductivity $K_{x}$/$K_{y}$/$K_{z}$, coefficient of thermal expansion $\alpha_{x}$/$\alpha_{y}$/$\alpha_{z}$, and mass density.

The calculation of the equivalent Young’s modulus in X direction $E_{x}$ (Figure 4a) will be demonstrated as an example. The corresponding model boundary conditions will be given in detail. The lower left corner of the model is defined as the origin, with the fix-origin constraint (x=0, y=0, z=0, $U_{x}=U_{y}=U_{z}=0$). For the two faces parallel to the YZ plane, all nodes on the left face have an X-displacement of zero (x=0, $U_{x}=0$), while those on the right face have a displacement of $D_{x1}$ ($x=L_{x}$, $U_{x}=D_{x1}$). For the two faces parallel to the XZ plane, all nodes on the front face have a Y-displacement of zero (y=0, $U_{y}=0$), while those on the back face have the same displacement change ($y=L_{y}$, $U_{y}=U_{y,\mathrm{couple}}$). For the two faces parallel to the XY plane, all nodes on the bottom face have a Z-displacement of zero (z=0, $U_{z}=0$), while those on the top face have the same displacement change ($z=L_{z}$, $U_{z}=U_{z,\mathrm{couple}}$). After setting the boundary conditions, the block-level model is solved and $E_{x}$ can be expressed as follows:

(1) $$\sigma_{x}=\frac{F_{x1}}{L_{y}L_{z}}$$

(2) $$\varepsilon_{x}=\frac{D_{x1}}{L_{x}}$$

(3) $$E_{x}=\frac{\sigma_{x}}{\varepsilon_{x}}$$

where $F_{x1}$ is the average reaction force in the X direction of all nodes on the left face; $D_{x1}$ is the average displacement in the X direction of all nodes on the right face; and $L_{x}$, $L_{y}$, $L_{z}$ are the lengths of the block-level model in X, Y, Z directions, respectively. At the same time, $\nu_{\mathrm{xy}}$ and $\nu_{\mathrm{xz}}$ can be expressed as follows:

(4) $$\varepsilon_{y}=\frac{D_{y1}}{L_{y}}$$

(5) $$\varepsilon_{z}=\frac{D_{z1}}{L_{z}}$$

(6) $$\nu_{\mathrm{xy}}=-\frac{\varepsilon_{y}}{\varepsilon_{x}}$$

(7) $$\nu_{\mathrm{xz}}=-\frac{\varepsilon_{z}}{\varepsilon_{x}}$$

where $D_{y1}$ is the average displacement in the Y direction of all nodes on the front face, and $D_{z1}$ is the average displacement in the Z direction of all nodes on the top face. Similarly, with corresponding boundary conditions, $E_{y}$, $E_{z}$, and $\nu_{\mathrm{yz}}$ can also be extracted (Figure 4b,c).

The calculation of the equivalent shear modulus $G_{\mathrm{xy}}$ (Figure 4d) will also be demonstrated as an example. For the two faces parallel to the XZ plane, all the nodes on the front face are fixed in the X, Y, and Z directions (y=0, $U_{x}=U_{y}=U_{z}=0$), while on the back face, nodes are fixed in the Y and Z directions and move by $D_{x2}$ in the X direction ($y=L_{y}$, $U_{x}=D_{x2}$, $U_{y}=U_{z}=0$). For the two faces parallel to the XY plane, all nodes on both the top ($z=L_{z}$, $U_{z}=0$) and bottom faces (z=0, $U_{z}=0$) are fixed in the Z direction. After setting the boundary conditions, the block-level model is solved and $G_{\mathrm{xy}}$ can be expressed as follows:

(8) $$\sigma_{\mathrm{xy}}=\frac{F_{x2}}{L_{x}L_{z}}$$

(9) $$\varepsilon_{\mathrm{xy}}=\frac{D_{x2}}{L_{y}}$$

(10) $$G_{\mathrm{xy}}=\frac{\sigma_{\mathrm{xy}}}{\varepsilon_{\mathrm{xy}}}$$

where $F_{x2}$ is the average reaction force in the X direction of all nodes on the front face, and $D_{x2}$ is the displacement in the X direction of all nodes on the front face. Similarly, with corresponding boundary conditions, $G_{\mathrm{xz}}$ and $G_{\mathrm{yz}}$ can also be extracted (Figure 4e,f).

For the calculation of the equivalent thermal conductivity $K_{x}$ (Figure 4g), the temperature at all nodes on the left face is set to $T_{1}$ (x=0, $T=T_{1}$), and the temperature at all nodes on the right face is set to $T_{2}$ ($x=L_{x}$, $T=T_{2}$). The top, bottom, front, and back faces are all considered adiabatic. After solving the model, $K_{x}$ can be expressed as follows:

(11) $$K_{x}=\frac{q_{x}L_{x}}{T_{1}-T_{2}}$$

where $q_{x}$ is the heat flux density in the X direction of all nodes on the front face. Similarly, with corresponding boundary conditions, $K_{y}$ and $K_{z}$ can also be extracted (Figure 4h,i).

For the calculation of the equivalent CTE $\alpha_{x}$, $\alpha_{y}$ and $\alpha_{z}$ (Figure 4j), boundary conditions are applied as follows: all nodes on the left face are fixed in the X direction (x=0, $U_{x}=0$), those on the front face are fixed in the Y direction (y=0, $U_{y}=0$), and those on the bottom face are fixed in the Z direction (z=0, $U_{z}=0$). To allow for orthotropic expansion, displacement is constrained to be equal (but not fixed to zero) for all nodes on the right face in the X direction ($x=L_{x}$, $U_{x}=U_{x,\;\mathrm{couple}}$), on the back face in the Y direction ($y=L_{y}$, $U_{y}=U_{y,\;\mathrm{couple}}$), and on the top face in the Z direction ($z=L_{z}$, $U_{z}=U_{z,\;\mathrm{couple}}$). A unit temperature difference ΔT is applied to all nodes. After solving the model, $\alpha_{x}$, $\alpha_{y}$, and $\alpha_{z}$ can be expressed as follows:

(12) $$\alpha_{x}=\frac{\Delta L_{x}}{L_{x}}$$

(13) $$\alpha_{y}=\frac{\Delta L_{y}}{L_{y}}$$

(14) $$\alpha_{z}=\frac{\Delta L_{z}}{L_{z}}$$

where $\Delta L_{x}$ is the average displacement change in the X direction at all nodes on the right face, $\Delta L_{y}$ is the average displacement change in the Y direction at all nodes on the back face, and $\Delta L_{z}$ is the average displacement change in the Z direction at all nodes on the top face. For the calculation of the equivalent mass density, a volume-weighted average method is used, which will not be elaborated on here.

#### 2.2.2. Die-Level Model

**Figure 5:**
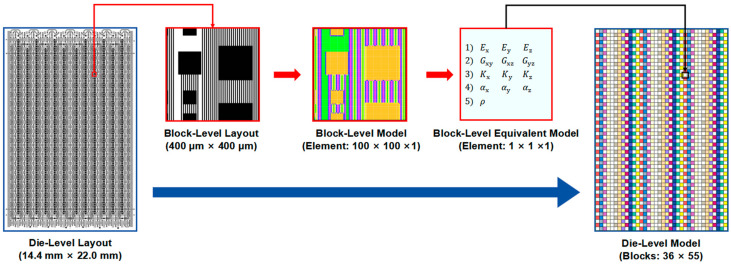
The method of forming equivalent die-level model.

**Figure 6:**
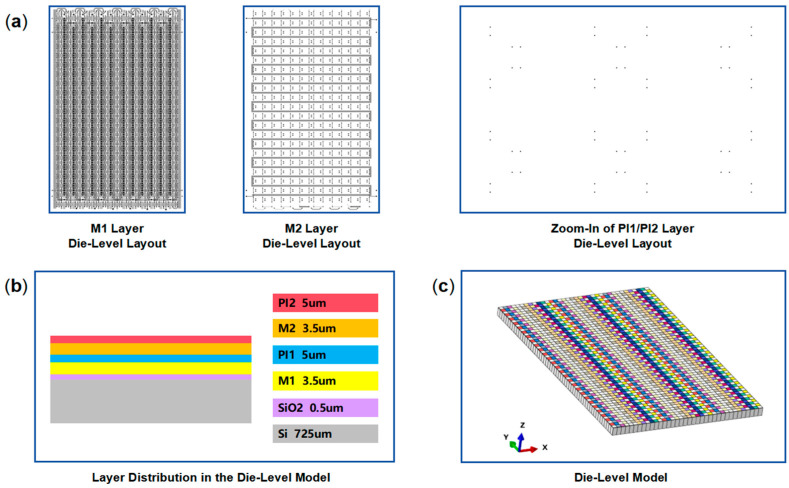
The details of the **e**quivalent die-level model. (**a**) Layer layouts of the M1, M2, PI1, and PI2 layers in the die-level region; (**b**) layer distribution and thicknesses in the die-level model; (**c**) die-level model in Abaqus.

The method for creating a die-level model is shown in Figure 5. First, a Python script (Python 3.12.4) is used to convert the RDL layout CAD file into a grayscale PNG image and further export a matrix of the grayscale value of each pixel, which also represents the metal volume fraction. Based on the matrix, a finite element model can be created for each block-level region within the die through Ansys APDL. Each block-level finite element model is then equivalently simplified using the RDL block equivalent material property calculation method (Figure 4) to yield a corresponding single-element block-level model. Finally, the die-level model is created by combining the single-element block-level models according to their position in the layout. Taking the M1 layer of the die-level region as an example, the plane dimensions of a die-level region are 14.4 mm × 22.0 mm, and the plane dimensions of the divided block-level regions are 400 µm × 400 µm. The corresponding block-level model in ANSYS APDL consists of 400 × 400 × 1 mesh elements. After solving for the orthotropic material properties, the equivalent block-level model reduces to a single mesh element. The M1 layer die-level model with 36 × 55 mesh elements is then created. The composition and details of each layer in the die-level model are shown in Figure 6. The die-level model containing Si, SiO_2_, two metal layers, and two via layers was built in Abaqus 2016 with 11,880 mesh elements.

#### 2.2.3. Wafer-Level Model

**Figure 7:**
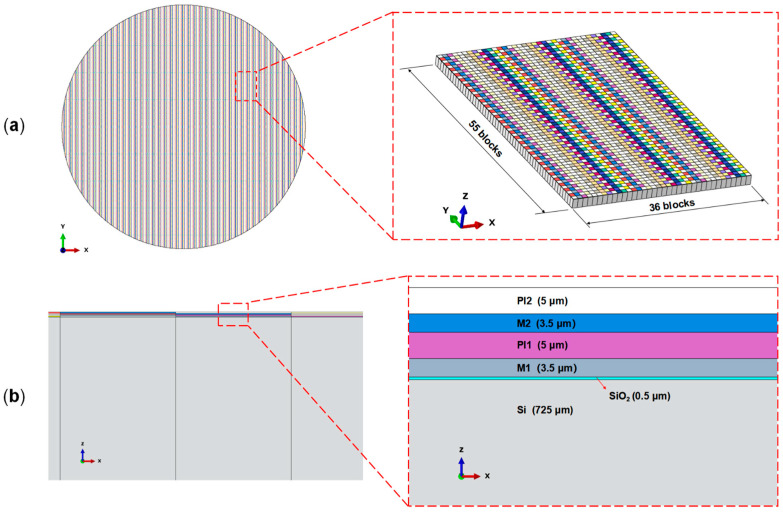
Equivalent FEM model of the 200 mm RDL wafer: (**a**) wafer-level equivalent model; (**b**) side view of the wafer-level equivalent model.

Figure 7 shows the wafer-level model, with the dummy region at the wafer edge also including patterned structures. Based on the feature sizes of each layer and the die arrangement on the wafer, an 8-inch RDL wafer-level model is created in Abaqus. The wafer-level model, consisting of M1, M2, PI1, and PI2 layers, is modeled using C3D8T (Coupled Temperature–Displacement) elements, comprising 1,411,694 elements.

### 2.3. Boundary Conditions

At the center of the wafer-level model, the boundary conditions are applied to constrain both displacement and rotation (U1 =U2 = U3 = UR1 = UR2 = UR3 = 0). The temperature boundary condition is applied by heating from 30 °C to 200 °C or 250 °C. These temperatures are chosen because 200 °C is the curing temperature for specific PI materials, while 250 °C is a typical process temperature for flip-chip (FC) bonding.

Another boundary condition for backgrinding and CMP is that the “removed” elements will be deactivated after corresponding steps in simulation, using the element birth and death technique.

### 2.4. Extraction of PI Material Properties

The RDL wafer consists of a silicon substrate with a thin silicon dioxide layer, copper for interconnects, and PI as the insulating material. Expect for PI, all materials are considered to be linear elastic.

**Figure 8:**
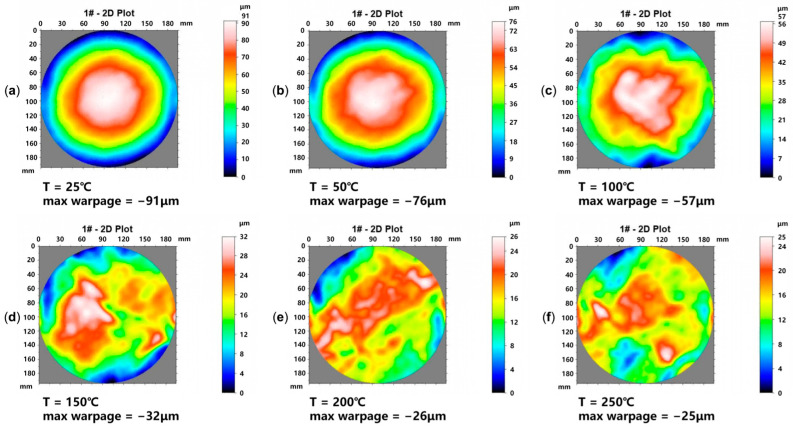
Thermal warpage test results of the 8-inch PI wafer at different temperature points. (**a**) Wafer warpage at 25 °C; (**b**) wafer warpage at 50 °C; (**c**) wafer warpage at 100 °C; (**d**) wafer warpage at 150 °C; (**e**) wafer warpage at 200 °C; (**f**) wafer warpage at 250 °C.

A PI wafer thermal warpage experiment was conducted to extract PI’s temperature-dependent CTE and reference temperature for simulation input. The PI wafer sample consists of a 725 µm thick 8-inch silicon wafer and a 5 µm thick PI layer (cured at 200 °C). The wafer was placed backside up (PI facing downward) and laid flat in the TDM Compact 3 system (based on the projection moiré principle). The temperature was increased from 25 °C to 250 °C to measure the thermal warpage, and the measurement results are shown in Figure 8. The Young’s modulus and Poisson’s ratio of the PI are known [24], while the CTE at different temperature points and the reference temperature need to be inversely deduced from the warpage measurement results. The Young’s modulus of PI was treated as a constant, and the fitting process adjusted the CTE of PI at different temperatures to ensure that the maximum warpage of the PI wafer in the simulation matched the measured value. The reason for this is that our simulations showed that the key factor affecting the Cu-PI RDL wafer warpage was the CTE mismatch between Cu and PI, rather than variations in the Young’s modulus of PI. For the thermal–mechanical behavior of wafer-level Cu-PI RDLs, it is more sensitive to the CTE of PI and less sensitive to the Young’s modulus. Therefore, the CTE values of PI were chosen to be fitted to develop a simulation method for predicting the Cu-PI RDL wafer warpage with high precision. In summary, we assumed a constant Young’s modulus for PI and adjusted its CTE across temperatures.

**Figure 9:**
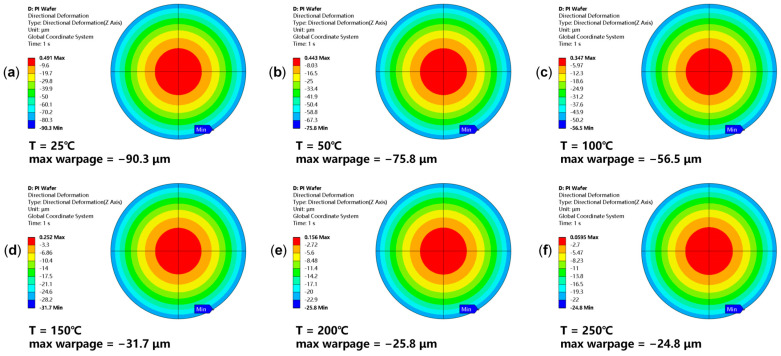
Thermal warpage simulation results of the 8-inch PI wafer at different temperature points. (**a**) Wafer warpage at 25 °C; (**b**) wafer warpage at 50 °C; (**c**) wafer warpage at 100 °C; (**d**) wafer warpage at 150 °C; (**e**) wafer warpage at 200 °C; (**f**) wafer warpage at 250 °C.

The reference temperatures of PI and silicon were set to 275 °C and 30 °C, respectively. Considering axial symmetry, a quarter model of the PI wafer is created for simulation, while the gravity factor (g = −9.81 m/s^2^) is also considered. The wafer’s maximum warpage at each temperature serves as an indicator. By comparing the simulated and measured maximum warpage values, the temperature-dependent CTE of PI can be determined. Figure 9 shows the simulation results of the PI wafer’s thermal warpage. As the measured and simulated values of the wafer maximum warpage at each temperature point show considerable agreement, the temperature-dependent CTE and reference temperature can be determined. Including the extracted PI parameters, the material properties used for the subsequent equivalent model are summarized in Table 1. The actual glass transition temperature (T_g_) of the PI used is 219 °C, which may contribute to the observed jump in the fitted CTE at higher temperatures.

## 3. Results and Discussion

### 3.1. Validation of Equivalent RDL Wafer Model

To validate the modeling and simulation method for predicting the thermo-mechanical behavior of the wafer-level Cu-PI RDL, the thermal warpage measurement results of 8-inch RDL wafer samples were used as a benchmark for a comparison with the simulation results. The RDL wafer sample (Figure 2b) comprises PI, Cu, Si, and SiO_2_ materials.

**Figure 10:**
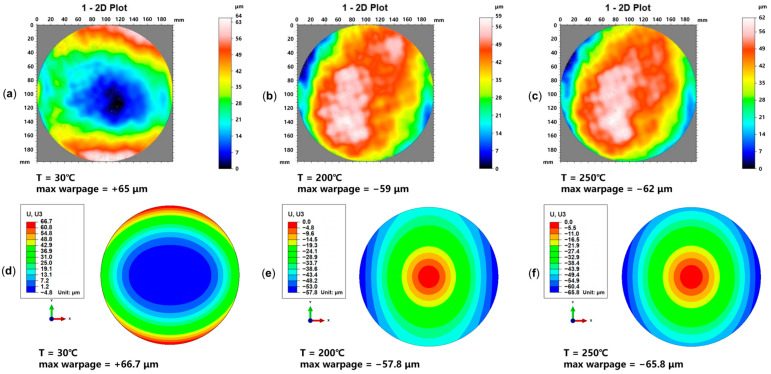
Measurement and simulation results of 8-inch RDL wafer warpage at typical temperature points. (**a**) Wafer warpage measurement results at 30 °C; (**b**) wafer warpage measurement results at 200 °C; (**c**) wafer warpage measurement results at 250 °C; (**d**) wafer warpage simulation results at 30 °C; (**e**) wafer warpage simulation results at 200 °C; (**f**) wafer warpage simulation results at 250 °C.

**Figure 11:**
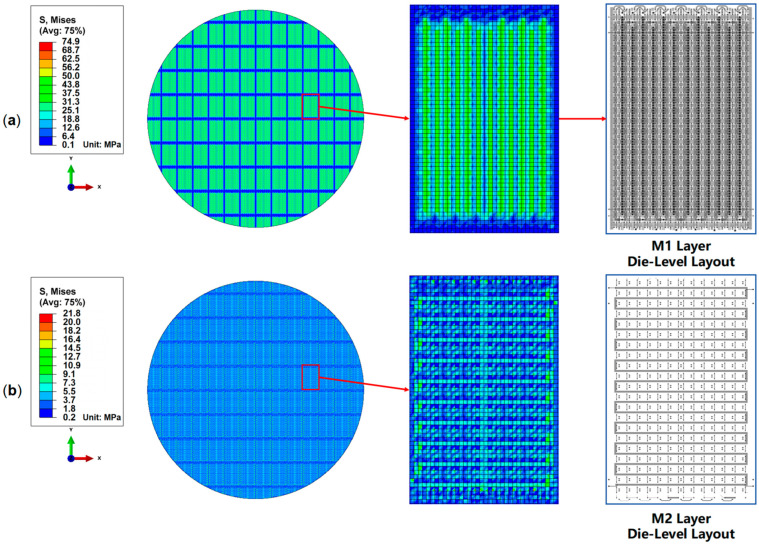
Von Mises stress distribution of the RDL at 200 °C. (**a**) Metal 1 layer; (**b**) metal 2 layer.

The RDL wafer sample was placed pattern-side up and flat in the TDM Compact 3 system, as the temperature increases from 30 °C to 250 °C during measurement. Figure 10a–c show the thermal warpage test results at typical temperatures of 30 °C, 200 °C, and 250 °C. The RDL wafer sample warpage changes from convex to concave. After multiple iterations of fitting, the wafer’s warpage-free temperature was determined to be 212 °C. Figure 10d–f show the warpage simulation results of the RDL wafer model at 30 °C, 200 °C, and 250 °C. At the three typical temperature points, the wafer’s maximum warpage error is 2.62%, 2.03%, and 6.13%, respectively. The error between the simulated maximum warpage values and the measured maximum warpage values is controlled within 10%, thereby validating the accuracy of the equivalent modeling approach. This model ran in an environment with an i7-14700kf CPU and 192GB memory; the solution time was about 15 min. Additionally, taking the simulation results at 200 °C as an example, Figure 11 shows the von Mises stress distributions in the M1 and M2 layers. The stress distribution implies the impact on the thermo-mechanical behavior introduced by the layout in each layer.

### 3.2. Impact of Increased RDLs on Wafer Warpage

**Figure 12:**
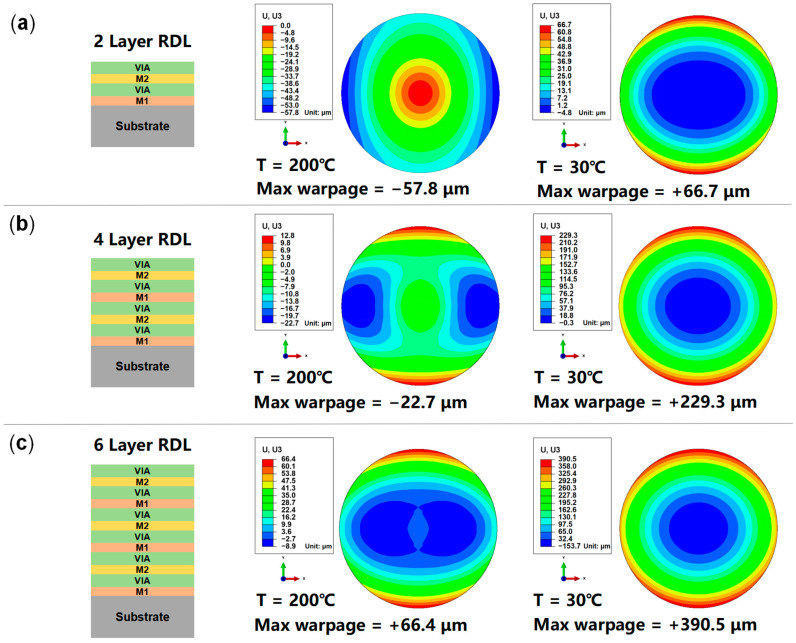
Prediction results of wafer warpage with different RDL counts. (**a**) Two-layer RDL; (**b**) four-layer RDL; (**c**) six-layer RDL.

The current RDL wafer sample features two layers of RDLs. To investigate the impact of increasing the number of RDLs on the warpage, equivalent models of wafers with four-layer RDLs and six-layer RDLs were also created. Since the curing temperature of PI is 200 °C, 30 °C and 200 °C were selected as typical temperatures to evaluate the warpage distribution of the equivalent wafer models. The model composition and corresponding prediction results are shown in Figure 12. As the number of RDLs increases, the wafer warpage at 200 °C initially decreases and then increases, with a gradual transition of the warpage pattern from concave to convex. On the other hand, at 30 °C, the wafer warpage continues to increase as the number of RDLs increases, with a consistent convex pattern.

### 3.3. Warpage Prediction of RDL Wafer at Subsequent Process Steps

**Figure 13:**
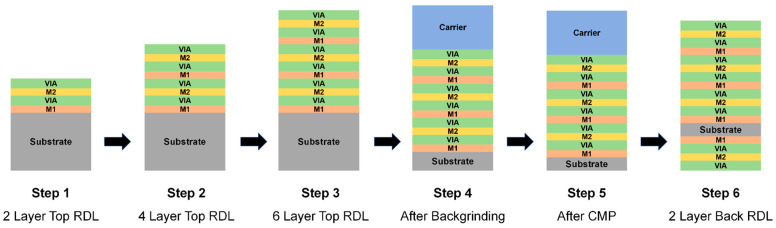
Process steps of the RDL wafer.

Subsequent processes for the RDL wafer include RDL build-up, substrate backgrinding and CMP, as illustrated in Figure 13. Based on the material parameters in Table 1, an equivalent model was created to investigate wafer warpage during these process steps and assess potential risks. Warpage predictions were conducted at three key points: after backgrinding, after CMP, and after adding two back RDLs. During backgrinding and CMP, a 550 μm thick BF33 glass wafer was added as a carrier.

**Figure 14:**
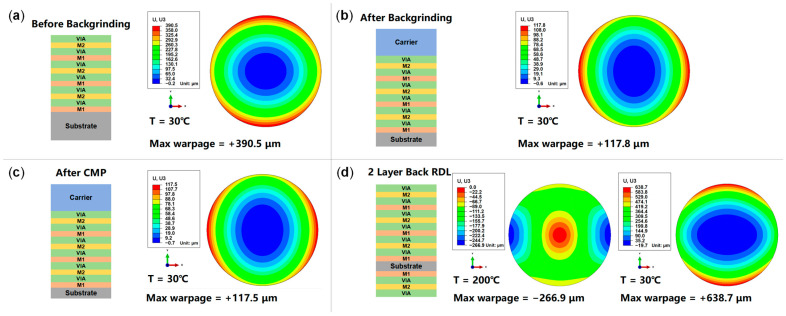
Prediction results of wafer warpage in different subsequent process steps: (**a**) before backgrinding; (**b**) after backgrinding; (**c**) after CMP; (**d**) adding two-layer back RDLs.

Taking the case of a silicon substrate thinned to 200 µm as an example, the predicted wafer warpage for the subsequent process steps is shown in Figure 14. It is evident that even though the silicon substrate is thinned from 725 µm to 200 µm, the wafer warpage is effectively controlled at an acceptable level by adding a glass carrier. The subsequent CMP has almost no effect on the warpage. Adding two layers of RDLs on the back side will balance out some stress, reducing the warpage to −266.9 μm at 200 °C and finally increasing it to +638.7 μm at 30 °C, presenting a convex pattern.

**Figure 15:**
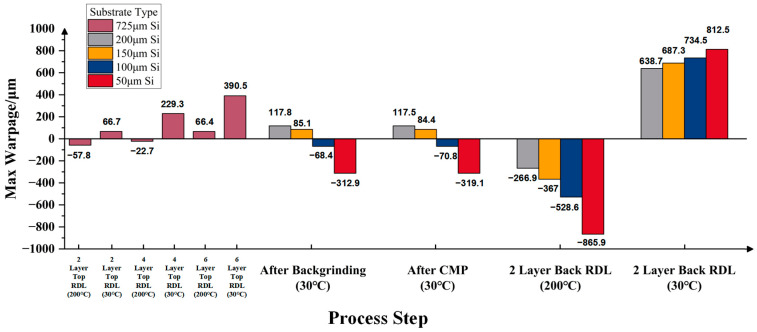
Prediction results of wafer warpage with different silicon substrate thicknesses.

Figure 15 presents the predicted warpage of the RDL wafers on silicon substrates with four post-thinning thicknesses—200 μm, 150 μm, 100 μm, and 50 μm—across various process steps. With the increase in top-side RDL build-up layers, the wafer warpage gradually increases, showing a convex pattern. After backgrinding and CMP, warpage decreases; wafers with 200 μm and 150 μm silicon substrates still exhibit a convex pattern, while those with 100 μm and 50 μm substrates show a concave pattern. After bottom-side RDL build-up, the maximum warpage at 200 °C increases significantly, with all wafers showing a concave pattern, which transforms into a convex pattern upon cooling to room temperature. The thinnest substrate (50 μm) consistently results in the highest maximum warpage among the four thickness conditions.

To ensure reliability, the maximum warpage of a 12-inch wafer should not exceed 1 mm. Furthermore, for high-yield manufacturing, the warpage should be limited to within 0.5 mm [27]. Based on this benchmark and scaling down to 8 inches, the corresponding limits should be 666 μm for essential reliability and 333 μm for a high yield. According to the simulation results, among the tested thicknesses, the 8-inch RDL wafer with a 200 μm thick silicon substrate satisfies the reliability requirement but does not meet the warpage requirement for high yields.

### 3.4. Impact of Substrate on Wafer Warpage

In previous studies, the substrate material was silicon with a silicon dioxide layer. To better match the actual application of RDL interposers, the substrate is replaced with equivalent TSV and TGV materials to predict the warpage after backgrinding, CMP, and the fabrication of two RDLs on the backside. As the equivalent material property extraction method for TSV and TGV was established in our earlier study [28], it will not be elaborated on again here. The equivalent material parameter extraction method for TSV and TGV is similar to that shown in Figure 5. The material parameters of glass are the same as the carrier [29], which has been shown in Table 1. The TSV and TGV structures in the substrate have a diameter of 10 µm and a pitch of 80 µm. For TSVs/TGVs with a uniform diameter of 10 µm and varying heights of 50 µm, 100 µm, 150 µm, and 200 µm, the corresponding aspect ratios are 5:1, 10:1, 15:1, and 20:1, respectively. The extracted equivalent TSV and TGV substrate parameters are listed in Table 2 and Table 3, respectively.

**Figure 16:**
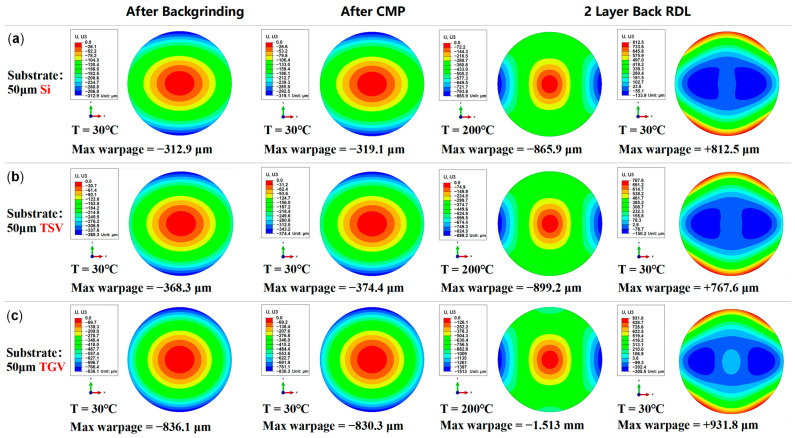
Warpage prediction contour plots for wafers with different substrate materials after backgrinding, CMP, and the addition of two back RDLs. (**a**) Silicon substrate with a thickness of 50 µm. (**b**) Equivalent TSV substrate with a thickness of 50 µm. (**c**) Equivalent TGV substrate with a thickness of 50 µm.

When the substrate thickness is 50 µm, the corresponding wafer warpage results for the silicon, equivalent TSV, and equivalent TGV substrates are summarized in Figure 16. It can be concluded that after backgrinding, CMP, and adding two back RDLs, the maximum warpage of the RDL wafer with an equivalent TSV substrate is smaller than that of the RDL wafer with a silicon substrate. In contrast, the RDL wafer with an equivalent TGV substrate shows a significantly greater maximum warpage. This is considered reasonable as the modulus of glass (64 GPa) is much lower than the modulus of silicon (131 GPa), which makes the equivalent TGV modulus lower than the equivalent TSV modulus. As a result, under the same stress, the RDL wafer with an equivalent TGV substrate shows more deformation, leading to significantly greater warpage than the wafers with silicon or equivalent TSV substrates.

**Figure 17:**
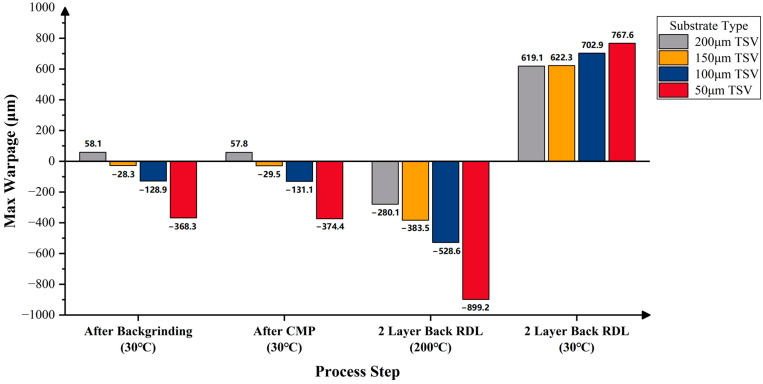
Prediction results of wafer warpage with different equivalent TSV substrate thicknesses.

**Figure 18:**
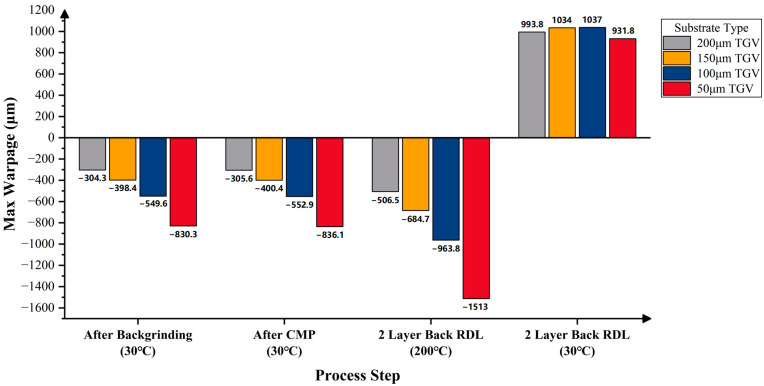
Prediction results of wafer warpage with different equivalent TGV substrate thicknesses.

Similarly, the predicted warpage results for RDL wafers with post-thinning substrate thicknesses of 200 μm, 150 μm, 100 μm, and 50 μm are shown in Figure 17 and Figure 18, where the substrates are equivalent TSV and TGV substrates, respectively. After completing the entire process flow, the maximum warpage of the RDL wafers with equivalent TSV substrates at thicknesses of 200 μm and 150 μm meets the reliability requirement. In contrast, the maximum warpage of the RDL wafers with equivalent TGV substrates exceeds the reliability limit in all cases, indicating further room for optimization.

It is worth mentioning that after the entire process, the maximum warpage of the wafers with silicon substrates and equivalent TSV substrates increases as the substrate thickness decreases. For the wafers with equivalent TGV substrates, the maximum warpage first increases and then decreases as the substrate thickness decreases. The maximum warpage of the RDL wafer with a 50 μm thick substrate is even smaller than that of the wafer with a 200 μm thick substrate.

The maximum allowable warpage for an 8-inch wafer is 666 μm for reliability and 333 μm for high yield. When the TSV wafer is thinned to 200 μm or 150 μm, the final wafer warpage remains below 666 μm, thus meeting the reliability requirement (Figure 17). However, if the TSV wafer is further thinned to 100 μm or 50 μm, the warpage will exceed 666 μm and fail to meet the reliability requirement. For TGV wafers, all cases with a substrate thickness of 200 μm or less could result in more than 666 μm final warpages, indicating that none of these configurations satisfy the reliability requirement (Figure 18).

Therefore, a carrier material with a higher Young’s modulus than glass is recommended for the backgrinding and CMP processes to improve yield and reliability. In addition, when adding back-side RDLs, an additional top-side carrier layer should be applied to help balance the stress and reduce wafer warpage.

### 3.5. Neural Network Model for Wafer Warpage Prediction

**Figure 19:**
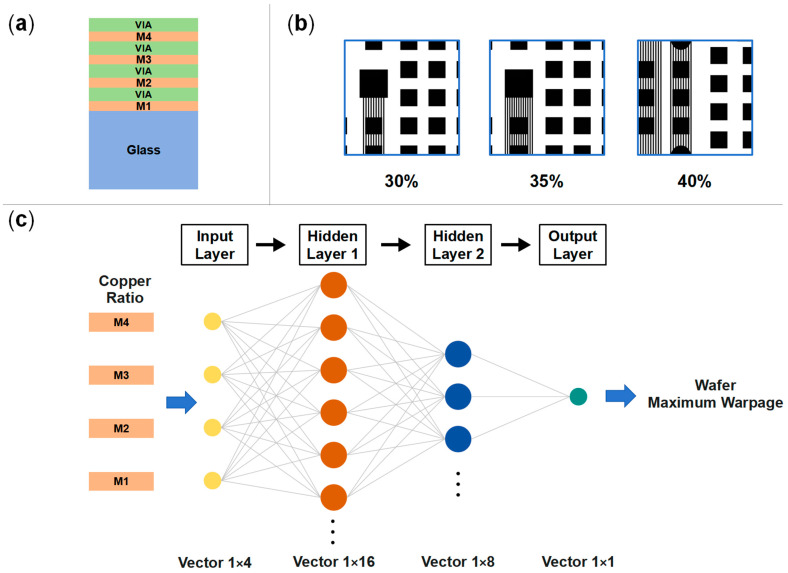
Establishment of neural network model for predicting warpage. (**a**) Wafer-level model with four RDLs and 725 μm thick glass substrate. (**b**) Block-Level RDL layouts for different copper proportions. (**c**) FCNN model for prediction of wafer maximum warpage.

The purpose of this section is to demonstrate how to use the simulation method for improving manufacturability. Therefore, warpage optimization was performed by adjusting the copper ratio in the RDLs during the four-layer RDL build-up process. A representative case was selected in this paper for conceptual demonstration. To demonstrate the extended application of the proposed method, a 725 μm thick glass wafer with four RDLs was analyzed as a case (Figure 19a). An ANN model was created to establish the correlation between the maximum wafer warpage and the copper ratio combinations of each RDL, aiming to extract potential optimization schemes for copper ratio design. The copper ratio of a single layer is allowed to be 30%, 35%, or 40%, as the initial value is set to be 35% for each RDL. The corresponding block-level layout patterns for the three copper ratios are illustrated in Figure 19b. A fully connected neural network (FCNN) was employed, consisting of two hidden layers with Rectified Linear Unit (ReLU) activation functions. The input layer represents the copper ratio of the four RDLs, and the output layer predicts the maximum wafer warpage.

**Figure 20:**
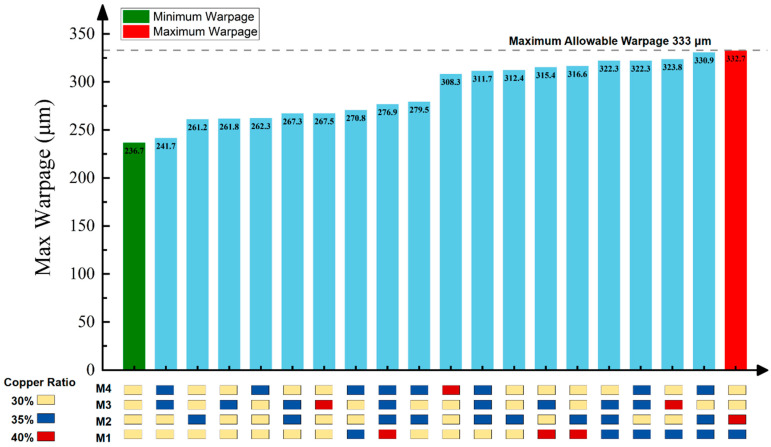
Predicted warpage results for different RDL copper ratio combinations using ANN model.

Considering the high yield requirement, the maximum allowable warpage for this wafer is 333 μm. Based on limited simulation data sets, the trained ANN model was used to identify the copper ratio combinations that meet this warpage requirement. The results are summarized in Figure 20. It can be seen that 20 optimization combinations of copper ratios are given to ensure that the wafer warpage meets the requirement. Starting from a wafer with an initial copper ratio of 35% in all RDLs, the ANN model identified 20 optimized copper ratio combinations that satisfied the warpage requirement. The minimum predicted warpage of 236.7 μm occurred when all four RDLs had a copper ratio of 30%. In contrast, the maximum warpage of 332.7 μm was observed when the copper ratios were 35%, 40%, 30%, and 30% for M1, M2, M3, and M4, respectively. The results demonstrated that, starting from an initial configuration of a 35% copper ratio in all RDLs, the proposed ANN-based approach can effectively guide multi-layer copper ratio optimization to control wafer warpages. It should be claimed that this method can be extended to wafers with any number of RDLs and any concerned process stage.

## 4. Conclusions

This study presented an efficient and accurate simulation method for predicting the warpage of wafer-level Cu-PI RDLs during manufacturing. A cross-scale wafer-level equivalent model was established for simulation analysis based on temperature-dependent PI material properties extracted through inverse fitting. The consistency between the simulated wafer warpage and experimental measurements validated the accuracy of the extracted PI properties, indicating that the cross-scale equivalent model effectively predicted the thermo-mechanical behavior of the Cu-PI RDL wafers under typical process temperatures. The simulation achieved an absolute prediction error below 10%, while significantly reducing the model complexity and mesh count. The simulation maintained a low prediction error while significantly reducing the model complexity and mesh count. The effects of RDL build-up, wafer backgrinding, CMP, and different substrate types on wafer warpage were also evaluated, providing insights for risk assessment and reliability evaluation in future Cu-PI RDL fabrication on TSV and TGV wafer-level substrates. Furthermore, an ANN model was created as an example to reveal the correlation between the maximum wafer warpage and the copper ratio of each RDL, providing guidance for wafer warpage optimization through copper ratio adjustments.

## Figures and Tables

**Table 1:** Material properties for simulation analysis of RDL wafer model.

Materials	Young’s Modulus (GPa)	Poisson’s Ratio	Thermal Expansion Coefficient (ppm/°C)	Thermal Conductivity (W/m·K)	Density (kg/m^3^)
Si [25]	131	0.28	2.6	145.87	2.329
SiO_2_ [18]	73	0.17	0.55	1.1	2.300
Cu [26]	130	0.34	16.4	401	8.960
PI	2.5	0.35	54 (25 °C) 50 (50 °C) 47 (100 °C) 35 (150 °C) 44.5 (200 °C) 122 (250 °C)	0.12	1.420
Glass	64	0.20	3.25	1.2	2.230

**Table 2:** Material properties of equivalent TSV substrate with different thicknesses for simulation analysis.

Materials	Young’s Modulus (GPa)	Poisson’s Ratio	Shear Modulus (GPa)	CTE (ppm/°C)	Thermal Conductivity (W/m·K)	Density (kg/m^3^)
Equivalent TSV (h = 50 μm)	E_x_ = 130.997868	ν_xy_ = 0.280822	G_xy_ = 51.138030	α_x_ = 2.789632	K_x_ = 147.549900	2.410
	E_y_ = 130.997868	ν_xz_ = 0.281142	G_xz_ = 51.138315	α_y_ = 2.789632	K_y_ = 147.549900	
	E_z_ = 130.994392	ν_yz_ = 0.281146	G_yz_ = 51.138314	α_z_ = 2.873377	K_z_ = 149.000899	
Equivalent TSV (h = 100 μm)	E_x_ = 130.998558	ν_xy_ = 0.280829	G_xy_ = 51.138030	α_x_ = 2.791227	K_x_ = 147.549900	2.410
	E_y_ = 130.998558	ν_xz_ = 0.280973	G_xz_ = 51.138315	α_y_ = 2.791227	K_y_ = 147.549900	
	E_z_ = 130.994393	ν_yz_ = 0.280975	G_yz_ = 51.138315	α_z_ = 2.828727	K_z_ = 149.000899	
Equivalent TSV (h = 150 μm)	E_x_ = 130.998788	ν_xy_ = 0.280831	G_xy_ = 51.138030	α_x_ = 2.791696	K_x_ = 147.549899	2.410
	E_y_ = 130.998788	ν_xz_ = 0.280931	G_xz_ = 51.138315	α_y_ = 2.791696	K_y_ = 147.549899	
	E_z_ = 130.994393	ν_yz_ = 0.280930	G_yz_ = 51.138315	α_z_ = 2.817442	K_z_ = 149.000899	
Equivalent TSV (h = 200 μm)	E_x_ = 130.998903	ν_xy_ = 0.280832	G_xy_ = 51.138030	α_x_ = 2.791925	K_x_ = 147.549899	2.410
	E_y_ = 130.998903	ν_xz_ = 0.280899	G_xz_ = 51.138315	α_y_ = 2.791925	K_y_ = 147.549899	
	E_z_ = 130.994392	ν_yz_ = 0.280898	G_yz_ = 51.138315	α_z_ = 2.809058	K_z_ = 149.000899	

**Table 3:** Material properties of equivalent TGV substrate with different thicknesses for simulation analysis.

Materials	Young’s Modulus (GPa)	Poisson’s Ratio	Shear Modulus (GPa)	CTE (ppm/°C)	Thermal Conductivity (W/m·K)	Density (kg/m^3^)
Equivalent TGV (h = 50 μm)	E_x_ = 64.494801	ν_xy_ = 0.201541	G_xy_ = 26.838356	α_x_ = 3.467406	K_x_ = 1.229639	2.312
	E_y_ = 64.494801	ν_xz_ = 0.201408	G_xz_ = 26.857501	α_y_ = 3.467406	K_y_ = 1.229639	
	E_z_ = 64.831363	ν_yz_ = 0.201373	G_yz_ = 26.857501	α_z_ = 3.703234	K_z_ = 6.106258	
Equivalent TGV (h = 100 μm)	E_x_ = 64.494989	ν_xy_ = 0.201543	G_xy_ = 26.838356	α_x_ = 3.468674	K_x_ = 1.229639	2.312
	E_y_ = 64.494989	ν_xz_ = 0.201344	G_xz_ = 26.857501	α_y_ = 3.468674	K_y_ = 1.229639	
	E_z_ = 64.831363	ν_yz_ = 0.201327	G_yz_ = 26.857502	α_z_ = 3.659611	K_z_ = 6.106258	
Equivalent TGV (h = 150 μm)	E_x_ = 64.495051	ν_xy_ = 0.201544	G_xy_ = 26.838356	α_x_ = 3.469015	K_x_ = 1.229639	2.312
	E_y_ = 64.495051	ν_xz_ = 0.201319	G_xz_ = 26.857501	α_y_ = 3.469015	K_y_ = 1.229639	
	E_z_ = 64.831363	ν_yz_ = 0.201328	G_yz_ = 26.857502	α_z_ = 3.650259	K_z_ = 6.106258	
Equivalent TGV (h = 200 μm)	E_x_ = 64.495082	ν_xy_ = 0.201544	G_xy_ = 26.838356	α_x_ = 3.469169	K_x_ = 1.229639	2.312
	E_y_ = 64.495082	ν_xz_ = 0.201308	G_xz_ = 26.857501	α_y_ = 3.469169	K_y_ = 1.229639	
	E_z_ = 64.831363	ν_yz_ = 0.201317	G_yz_ = 26.857502	α_z_ = 3.641562	K_z_ = 6.106258	

## Data Availability

The data presented in this study are available on request from the corresponding author. The data are not publicly available due to privacy.
